# Properties of Dual-Crosslinked Collagen-Based Membranes as Corneal Repair Material

**DOI:** 10.3390/jfb14070360

**Published:** 2023-07-10

**Authors:** Lulu Wang, Yuehai Peng, Wenfang Liu, Li Ren

**Affiliations:** 1 Henan Provincial People’s Hospital, Henan Eye Hospital, Zhengzhou University People’s Hospital, Zhengzhou 450003, China; doctwang@zzu.edu.cn; 2 National Engineering Research Center for Tissue Restoration and Reconstruction, Guangzhou 510006, China; pengyuehaizz@163.com (Y.P.); liuwenfang2019@163.com (W.L.); 3 Guangzhou Proud Seeing Biotechnology Co., Ltd., Guangzhou 510623, China; 4 School of Biology and Biological Engineering, South China University of Technology, Guangzhou 510006, China; 5 School of Materials Science and Engineering, South China University of Technology, Guangzhou 510006, China

**Keywords:** collagen membrane, corneal repair, corneal regeneration

## Abstract

Corneal disease has become the second leading cause of blindness in the world. Corneal transplantation is currently considered to be one of the common treatments for vision loss. This paper presents a novel approach utilizing dual-crosslinked membranes composed of polyrotaxane multiple aldehydes (PRAs), 1-ethyl-3-(3-dimethylaminopropyl) carbodiimide (EDC), and N-hydroxysuccinimide (NHS) in the development process. Collagen was crosslinked, respectively, by EDC/NHS and PRAs to form stable amide bonds and imine groups. Through the formation of a double interpenetrating network, dual-crosslinked (Col-EDC-PRA) membranes exhibited enhanced resistance to collagenase degradation and superior mechanical properties compared to membranes crosslinked with a single crosslinker. Furthermore, Col-EDC-PRA membranes display favorable light transmittance and water content characteristics. Cell experiments showed that Col-EDC-PRA membranes were noncytotoxic and were not significantly different from other membranes. In a rabbit keratoplasty model, corneal stromal repair occurred at 5 months, evidenced by the presence of stromal cells and neo-stroma, as depicted in hematoxylin–eosin-stained histologic sections and optical coherence tomography images of the anterior segment. Moreover, there was no inflammation and corneal neovascularization, as well as no corneal rejection reaction in the surgical area. Overall, the results demonstrated that the dual-crosslinked membranes served effectively for corneal tissue regeneration after corneal defect.

## 1. Introduction

Corneal blindness is a major cause of vision loss, ranking second among debilitating diseases [1]. With the rapid growth of population, the number of the global population with corneal blindness is expected to reach 61 million in 2050, with even more individuals experiencing varying degrees of corneal impairment other than blindness [2]. While corneal transplantation is an established treatment modality for restoring corneal structure [3], it faces challenges due to legal, ethical, religious, and cultural limitations, resulting in a shortage of donors, especially in underdeveloped regions [4]. Therefore, there is a growing interest in developing strategies that mimic the natural corneal structure [5,6,7].

Currently, several types of implants are used in clinical practice, each with their own limitations. For example, acellular porcine corneal stromal donor material is widely used; usually, it is used as a transplant material to treat corneal defects or keratoconus crosslinked by riboflavin/UV [6]. However, it carries immunogenicity risks and offers poor optical reconstruction outcomes [8,9]. Amniotic membrane material, on the other hand, has poor mechanical strength and is primarily employed for corneal surface repair [10]. Artificial corneas made from synthetic polymers are prone to cause complications during application [11]. In addition, recent research has focused on 3D printing methods to create corneal repair materials [12]. Curvature-shaped corneal materials are prepared by mixing bioinks with different concentrations of GelMA and collagen [13]. Conversely, collagen, as the main component of the natural corneal stroma, exhibits excellent biocompatibility and makes a suitable scaffold material for corneal regeneration [6,14]. However, collagen derived from animals possesses drawbacks such as poor light transmittance and susceptibility to enzymatic degradation [15]. Therefore, the crosslinking of collagen is necessary to improve its physicochemical properties [16]. Previous studies have reported that the preparation of crosslinked membranes using 1-ethyl-3-(3-dimethylaminopropyl) carbodiimide (EDC) and N-Hydroxy succinimide (NHS) improves optical and mechanical performance [17]. However, the long-term stability of implants stabilized by EDC/NHS is limited, and these membranes are not suitable for the interrupted suture method commonly used in clinical procedures [18]. Riboflavin/UV light curing and polyethylene glycol have also recently been used as crosslinking methods with good biocompatibility; however, the mechanical strength that can be improved is limited for pure collagen and only suitable for suture-free repairs [19,20]. Furthermore, hydrogels dual-crosslinked by hyaluronic acid/NHS have been used to promote corneal re-epithelialization and wound healing but only for suture-free repairs [21,22]. Recently, our research group has employed polyrotaxane polyaldehydes and polyrotaxane multiple aldehydes (PRAs) as a crosslinker for collagen-based materials to improve collagen properties and promote corneal stroma remodeling. The poor light transmission performance of the material is due to the poor water solubility of polyrotaxane polyaldehyde molecules [23]. Therefore, PRA is considered more suitable for corneal repair materials [24]. On the basis of pure collagen being crosslinked by PRA, we selected EDC/NHS as the second crosslinking agent to prepare dual-crosslinked membranes for higher suture strength and stability. We characterized the water content, optical transparency, mechanical properties, enzymatic degradation, and cytotoxicity of PRA membranes, comparing them with EDC/NHS membranes to highlight their superior qualities. Furthermore, the corneal regeneration potential of these membranes was evaluated in an in vivo model of lamellar keratoplasty using New Zealand white rabbits.

## 2. Materials and Methods

### 2.1. Materials

Type I collagen (Pudao Lianxin Biotech, Co., Ltd., Guangzhou, China) was extracted from bovine tendon. Poly(ethylene glycol) (PEG, Mn = 2000), 1-Ethyl-3-(3-dimethylaminopropyl) carbodiimide (EDC), N-hydroxysuccinimide (NHS) triethylamine, and α-cyclodextrin were supplied from Aladdin (Shanghai, China), deionized water was obtained from the water purification system (Millipore S. A. S, Molsheim, France). Phosphate-buffered saline (PBS) was prepared from Gibco (Thermo Fisher Scientific, Waltham, MA, USA). Collagenase I from Clostridium histolyticum was obtained from Qiyun Biotechnology Co., Ltd. (Guangzhou, China) [25].

### 2.2. Preparation of Membranes

The synthesis of polyrotaxane multiple aldehydes (PRAs) was prepared as described in a previous work [18]. Type I collagen was dissolved in hydrochloric acid (pH = 2) at 4 °C and then air-dried in a clean mold to form an uncrosslinked collagen sample (Col). EDC and NHS were dissolved in deionized water with a concentration of 15 mg/mL. Then the two solutions were mixed with a mass ratio of Col:EDC:NHS = 6:1:1 at 4 °C. The mixed solutions were stirred using rotor-magnetic stirring at 4 °C for 24 h to make a homogeneous blend and crosslinked thoroughly. Collagen membranes crosslinked by EDC/NHS (Col-EDC) were prepared using the abovementioned method. For the preparation of the EDC/NHS and PRA crosslinked membranes, PRA solution with a concentration of 15 mg/mL was incorporated into the Col-EDC solution under magnetic stirring at 4 °C for 4 h. After crosslinking, the membranes were air-dried, sterilized, and stored at 25 °C for characterization. These collagen membranes were named Col-EDC-PRA.

### 2.3. Water Content

The water content of various samples was determined using a weighing method [26]. First, the weight of all dry samples was measured and recorded as W0 (n = 3). These samples were then immersed in normal saline, respectively, until equilibrium. After removing surface water from the samples with filter paper, the constant weight was measured and recorded as W1. Equilibrium water content was calculated using the following equation:

W = (W1 − W0)/W1 × 100%

### 2.4. Optical Properties

Light transmission was measured on a UV3802 ultraviolet-visible spectrophotometer (Shanghai UNICO, Shanghai, China) at 37 °C at a wavelength range of 380–800 nm. Before testing, membranes were saturated by immersing them in saline solution. They were then fixed in the specimen chamber of the spectrophotometer.

### 2.5. Mechanical Properties

The tensile test and suture strength test were characterized using a dynamic mechanical analyzer (DMA Q800) at 37 °C. Rectangular samples (width 5 mm, length 10 mm) were cut from the membranes and immersed in normal saline for 1 h. The stress–strain curve was obtained using the controlled force mode with a load velocity of 1 N/min.

During the suture strength test, one end of the rectangles was secured to the upper part of the metal jaw; then, the other end was pierced through using a 10-0 nylon suture (arrowheads) with tweezers and clamped in the lower metal jaw. The experimental process was carried out as previously described.

### 2.6. In Vitro Enzymatic Degradation

The method used for enzymatic degradation was described in our previous report [27]. Each sample was equilibrated in 5 mL PBS at 37 °C for 1 h, and the constant weight (W0) was measured. Collagenase (200 U/mg) was dissolved in PBS to obtain a solution concentration of 5 U/mL. The enzymatic degradation experiment was carried out in a shaker at 37 °C at a speed of 150 rpm. The collagenase solution was replaced every 8 h to maintain the collagenase activity. At specified time points, the surface of membranes was dried using filter paper and weighed (Wt). All membrane samples were tested in triplicate. The residual mass percentage was calculated using the following equation:

Residual mass (%) = Wt/W0 × 100%

### 2.7. In Vitro Biocompatibility

Rabbit corneal epithelial cells (RCECs) were obtained from the State Key Lab of Ophthalmology, Zhongshan Ophthalmic Center, Sun Yat-Sen University, Guangdong. RCECs were cultured in high glucose Dulbecco’s Modified Eagle’s Medium (DMEM; Gibco BRL, Waltham, MA, USA) with 10% fetal bovine serum (Gibco), penicillin (EGF; Gibco BRL) and streptomycin (HyClone, Logan, UT, USA). Cells were grown to confluency in 25 cm^2^ polystyrene tissue culture flasks at 37 °C in 5% CO_2_, and confluent cells were subcultured every 2–3 days using trypsinization with trypsin/EDTA solution. Col-EDC and Col-EDC-PRA were washed three times with PBS under aseptic conditions. The membranes were then transferred to a 48-well tissue culture plate (BD Biosciences, Tokyo, Japan). The control group consisted of RCECs cultured on tissue culture plates without collagen membranes. A specific volume of RCEC (5000 cells/mL) suspension was separately seeded onto the membranes. The culture medium was replaced every 2 days. At 1, 3, and 5 days, in culture, respectively, 100 μL of RCEC suspension was transferred to each well of 96-well, and tissue was added using a 10 μL CCK-8 solution (n = 3). The cytotoxicity of membranes was quantitatively determined by the CCK-8 (Dojindo, Kumamoto, Japan) assay using an optical density (OD) of 450 nm with a microplate reader. To qualitatively investigate the effect of Col-EDC and Col-EDC-PRA on cell behavior (adhesion and proliferation), RCECs were seeded at a density of 5 cells/mL on the surface of membranes. The culture medium was changed every other day.

### 2.8. Lamellar Keratoplasty in Rabbits

All experimental procedures conformed to the Association for Research in Vision and Ophthalmology (ARVO) Statement for the Use of Animals in Ophthalmic and Vision Research as well as local ethical rules. Four male New Zealand white rabbits, aged 1 year and weighing 2–2.5 kg, were used to create corneal injury models. Only the right eyes underwent lamellar keratoplasty, while another healthy rabbit cornea served as control. Before surgery, the membranes were immersed in saline water for over 1 h until saturation. All rabbits were anesthetized via pentobarbital sodium. The third eyelid was removed before surgery to prevent unnecessary wear on the membranes. Subsequently, a 6.5 mm diameter circular incision was made using a trephine. A lamellar dissection was then performed to remove the host epithelium and anterior stroma. The original corneal thickness was 350 ± 10 μm. The residual stromal bed thickness was measured as 150 ± 10 μm using a portable ultrasound pachymetry (Tomey, Nagoya, Japan). The membrane was secured to the corneal defect area using a continuous suture with 10–0 monofilament nylon sutures (Alcon, New Brunswick, NJ, USA). The medications administered post-lamellar keratoplasty included 0.1% Sodium hyaluronate eye drops (URSAPHARM Arzneimittel GmbH, Saarbrücken, Germany), Tobramycin dexamethasone eye drops (5 mL: Tobramycin 15 mg and dexamethasone 5 mg, Alcon, New Brunswick, NJ, USA) and 20% Calf blood deproteinizing extract eye gel (Shenyang Xingqi Pharmaceutical Co., Ltd., Shenyang, China). They were used four times a day. Tobramycin dexamethasone eye cream (5 mL: Tobramycin 15 mg and dexamethasone 5 mg, Alcon, New Brunswick, NJ, USA) was applied only at night. During the first seven days after surgery, penicillin (Shanxi Ruicheng Pharmaceutical Co., Ltd., Taiyuan, China) was injected into the muscle of the rabbit to reduce inflammation. In order to investigate the situation of the membranes in rabbit eyes, clinical examinations were conducted on days 7, 14, 2, 30, 90, and 180, using non-contact slit lamp microscopy to assess corneal optical clarity, conjunctival congestion, corneal edema, neovascularization, and anterior chamber inflammation. Sodium fluorescein staining was used to evaluate corneal epithelial integrity. Optical coherence tomography (OCT) was used to measure corneal thickness and observe tissue edema. At the end of the study, the animals were sacrificed 5 months after surgery, and the corneas were obtained for pathological examination and histology analysis.

### 2.9. Statistical Analysis

All data were presented as the mean ± standard deviation. Statistical analysis was performed using analysis of variance to determine significant differences among the groups. Statistical significance of *p* values was indicated as follows: * for *p* < 0.05, and n.s. for no significance. The sample size (n) for each statistical analysis was at least three.

## 3. Results

### 3.1. Mechanical, Optical, and Water Content Properties of Membranes

The water content of a material plays a crucial role in facilitating corneal nutrient transmission. In normal cornea, the water content ranges from 75 to 80%. The water content of corneal replacements can be influenced by the crosslinking reaction. Here, we measured the water content of Col-EDC and Col-EDC-PRA membranes. It is observed that both Col-EDC and Col-EDC-PRA membranes exhibited a decrease in water content compared to uncrosslinked collagen (Col). Notably, Col-EDC-PRA showed the lowest water content, measuring 62.40 ± 1.80%. Statistical analysis revealed a significant difference in water content between Col and Col-EDC, as well as between Col-EDC and Col-EDC-PRA. This decrease in water content can be attributed to the increased degree of crosslinking in the membranes.

**Figure 1:**
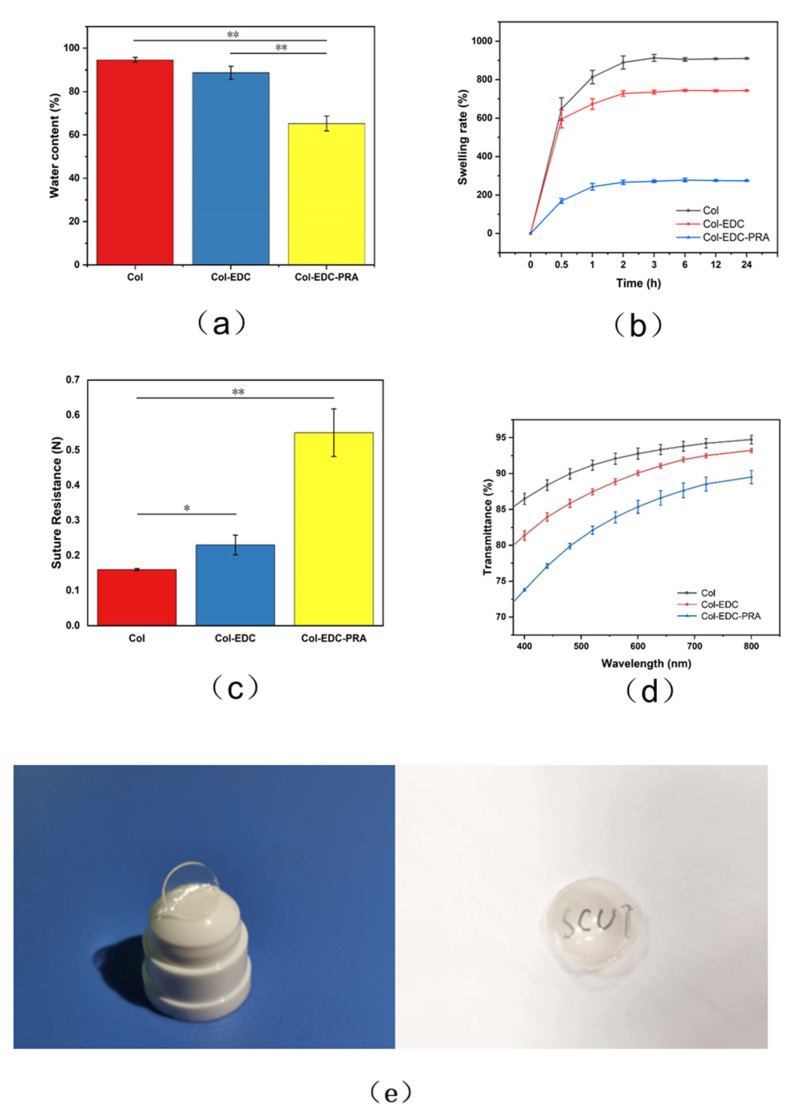
(**a**): Water content of Col, Col-EDC, and Col-EDC-PRA. (**b**): Swelling ratio of Col, Col-EDC, and Col-EDC-PRA at different time periods. (**c**): Mechanical property of Col, Col-EDC, and Col-EDC-PRA. (**d**): Optical property of Col, Col-EDC, and Col-EDC-PRA. (**e**) Image of Col-EDC-PRA sample. (*, 0.01 < *p* < 0.05; **, 0.001 < *p* < 0.01).

During surgical procedures, corneal implants need to possess exceptional resistance against rubbing and the forces exerted by nylon sutures. Therefore, the mechanical properties of corneal implants play an important role. The results presented in Figure 1c showed the suture strength of different materials. The suture strength of uncrosslinked collagen (Col) was measured to be 0.16 ± 0.02 N, while Col-EDC-PRA exhibited significantly higher suture strength, measuring 0.52 ± 0.08 N. The suture strength of EDC/NHS was also higher than that of Col, remaining at approximately 0.21 ± 0.05 N.

Light transmittance is another critical factor to consider. The natural human cornea exhibits higher light transmittance, reaching approximately 80% at a wavelength of 430 nm and nearly 100% above 500 nm. As shown in Figure 1d, the light transmittance of all the materials increased with an increasing wavelength. In the visible light wavelength range, both uncrosslinked collagen and Col-EDC displayed high light transmittance, surpassing 85% above the 500 nm wavelength in a wet state. There was no significant difference in light transmittance between these two materials. Although Col-EDC-PRA showed the lowest light transmittance, it still achieved nearly 85% at 780 nm. As shown in Figure 1e, the Col-EDC-PRA membrane still had good transparency when it was saturated with water. The letters below could be read easily. At the same time, the material could also maintain a good shape under wet conditions. so that it matched the curvature of the human cornea.

### 3.2. In Vitro Enzymatic Degradation and Cytotoxicity Assay

**Figure 2:**
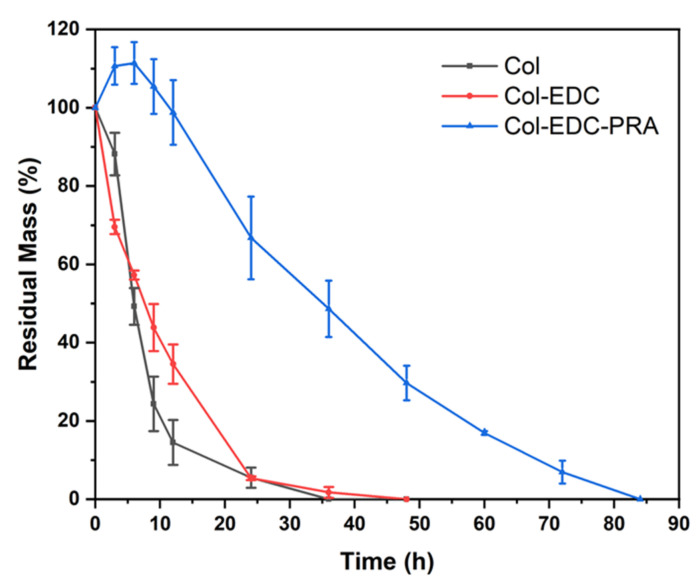
In vitro collagenase degradation of Col, Col-EDC, and Col-EDC-PRA.

The corneal membranes should provide long-term stability, as this is crucial for their role as scaffold materials, providing a conducive environment for corneal cell growth and cornea remodeling. As shown in Figure 2, both Col and Col-PRA experienced rapid degradation within 24 h, with no significant difference between the two. In comparison, the degradation curve of Col-EDC-PRA exhibited a relatively flat profile within 48 h, indicating a significantly slower degradation rate compared to the other groups.

**Figure 3:**
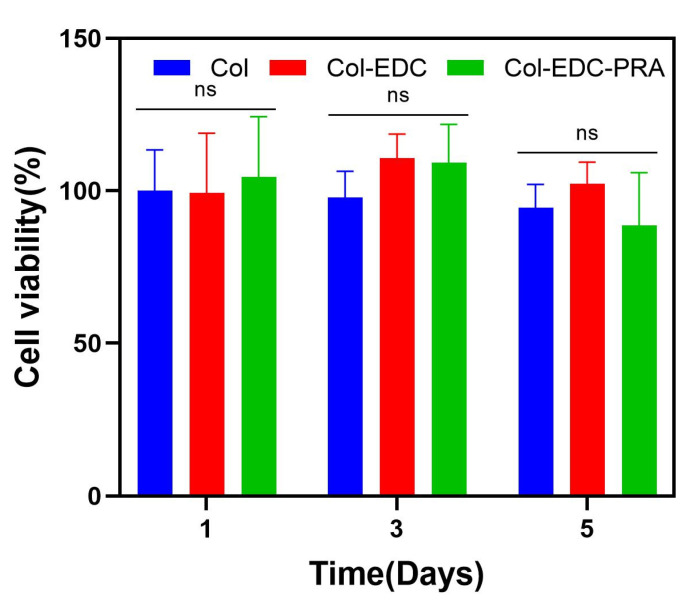
In vitro cell viability of Col, Col-EDC, and Col-EDC-PRA. (ns, 0.05 < *p*).

Ensuring excellent biocompatibility of corneal membranes is crucial for promoting faster wound healing and facilitating the formation of a protective barrier by the corneal epithelial cells. We evaluated the viability and proliferation of RCECs on Col-EDC and Col-EDC-PRA membranes, serving as a measure of their biocompatibility (Figure 3). The results revealed that both types of membranes exhibited excellent biocompatibility for the adhesion and proliferation of RCECs in vitro, with no significant difference compared to the control group. These results indicate that Col-EDC-PRA is noncytotoxic and can be safely utilized in animal experiments or clinic applications.

### 3.3. In Vivo Biocompatibility Assay

**Figure 4:**
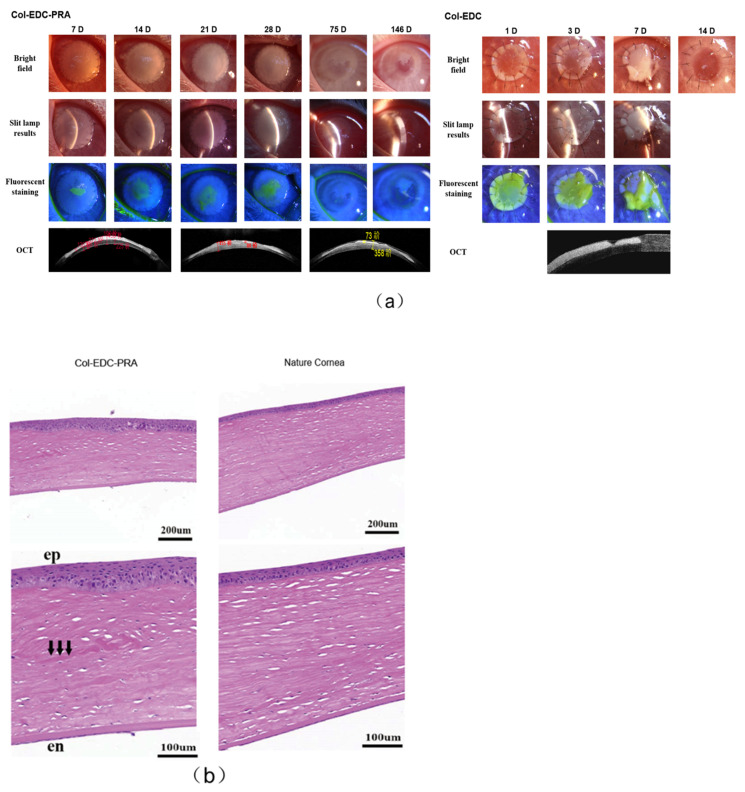
(**a**) Slit lamp biomicroscopy photographs and sodium fluorescein staining photographs of Col-EDC-PRA 146 days after operation. The OCT images were taken on days 14, 28, and 146, respectively. (**b**) Post-implantation histological examination (H&E staining) of Col-EDC-PRA and the H&E-stained section of the nature cornea at 146 days (scale bars as shown in the figure).

The therapeutic potential of Col-EDC-PRA in corneal regeneration was evaluated using a New Zealand white rabbit model of an anterior corneal lamellar injury. As observed in the results, Col-EDC-PRA gradually lost transparency after transplantation. The Col-EDC-PRA group exhibited accelerated re-epithelization, as evidenced by the fluorescein staining assay displayed in Figure 4a, where the absence of fluorescein dye retention indicated complete epithelialization in the area of interest. Notably, the operative area achieved 90% epithelialization within a period of 7 days, and the entire wound-healing process reached completion approximately 14 days after implantation. However, the implants in the control group (Col-EDC) were completely dissolved on day 14. The surgical sutures were removed after 28 days. No infectious or hemorrhagic complications occurred post-surgery. The suture experiment results indicated that Col-EDC-PRA possessed sufficient mechanical strength to withstand surgical sutures, providing a stable surface for RCEC migration. Rapid epithelialization is crucial for the recovery of corneal defects. In addition, re-epithelialization and de-epithelialization were observed in the prophase but stabilized over time, indicating that the adhesion between corneal epithelial and membrane graft was not strong, causing some epithelial cells to be rubbed off during rabbit blinking. On day 75, the transplanted cornea was completely epithelialized, and the wound was fully repaired. Over the course of the follow-up, the transparency of the material increased, allowing for a visualization of the iris texture and pupil through the graft on day 146.

Anterior segment optical coherence tomography (OCT), one of the most useful instruments in ophthalmic imaging, was used to measure corneal shape, corneal morphology, corneal thickness, and corneal curvature [28,29]. On day 7 after surgery, the anterior segment OCT showed that the Col-EDC-PRA adhered to the residual corneal stromal bed, with a clearly visible interface and higher density compared to the cornea. The overall thickness of the middle area of the cornea was 430 μm, while the thickness of the material was 130–190 μm. This demonstrated the presence of slight tissue edema between the stromal layers of the cornea. By day 75, the stromal distribution became more regular, with a density similar to that of the implanted Col-EDC-PRA. The interface between the residual corneal stroma and Col-EDC-PRA became indistinguishable, indicating complete fusion between the residual corneal stroma and Col-EDC-PRA. The corneal thickness at this time point was 353 μm. The original thickness of the cornea was restored and there was no tissue edema reaction and thinning caused by rapid local degradation in corneal stroma. Our final OCT on day 146 revealed no discernible interface between Col-EDC-PRA and the cornea stroma. Histology analysis further confirmed successful epithelial and stromal wound healing post-surgery (Figure 4b).

## 4. Discussions

**Figure 5:**
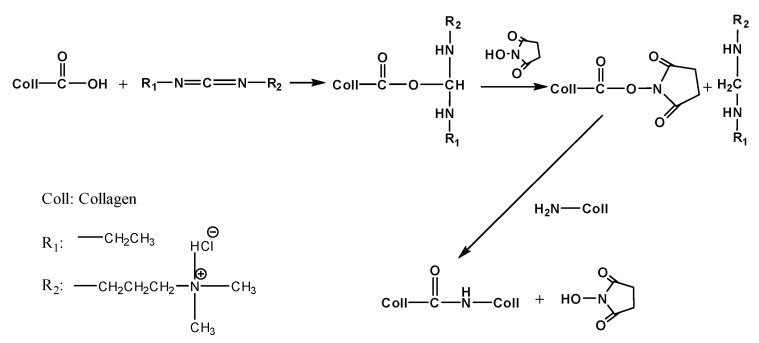
Scheme illustration for the reaction of collagen with EDC/NHS.

**Figure 6:**
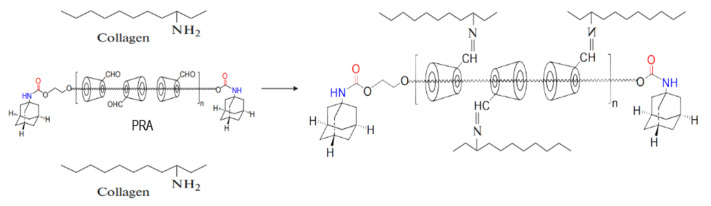
Scheme illustration for the reaction of collagen with PRA.

Type I collagen is the main component of the human cornea, and type I collagen scaffolds have attracted more and more attention as corneal repair materials. Collagen is rich in glycine (Gly), proline, and hydroxyproline, formed by a typical (Gly–X–Y) structure. The three peptide chains are connected by lateral covalent crosslinks and arranged in a helical manner. The amino and carboxyl groups on the side chains of the collagen molecules are easily reacted using chemical crosslink agents. The carboxyl groups of glutamic acid and aspartic acid are directly activated by EDC/NHS to form active esters, which are then crosslinked by free amino groups or hydroxyl groups of lysine to form amide bonds (Figure 5). The aldehyde group of PRAs can be crosslinked by the amino group of collagens to form a stable Schiff base. At the same time, PRA can effectively regulate the growth and assembly of collagen fibers through hydrogen bond interactions (Figure 6). Therefore, collagen–collagen segments and collagen–PRA segments together form a dual crosslinked network. The result of the mechanical properties could reach above 0.52 N. This is more applicable to the surgical requirements of deep lamellar layers compared to single crosslinked collagen membranes.

An in vitro enzymatic degradation experiment confirmed that Col-EDC-PRA effectively prevented collagenase from hydrolyzing the helical structure of the native collagen, due to the presence of a double crosslink network. Such degradation properties of Col-EDC-PRA aligned well with the denser multilayered lamella structure, which is known to be beneficial for corneal tissue regeneration. Moreover, the higher degree of the crosslinking of the material, the lower the swelling rate. This makes it difficult for collagenase to penetrate the middle of the material. Consequently, the dual-crosslinked membranes exhibited favorable transparency, with any reduction in the swelling rate contributing to the higher transmittance.

During in vivo lamellar keratoplasty, a reduction in transparency may be attributed to the presence of saline in tears, as a similar result was also observed in vitro, where the collagen membrane was rehydrated with normal saline during material preparation. The dense lamellar structure and crosslinker-regulated collagen fibril spacing of Col-EDC-PRA limited its water update and resulted in much closer fiber spacing. The implant still maintained a complete circular shape 75 days after lamellar keratoplasty, which is longer than the animal experiments in the group study [23,24]. This proved that the stability of the double crosslinked collagen membrane was better. At 146 days, part of the material could be seen in the bright field as cloudy and remained in the corneal stroma. This can affect the optical pathway and the patient’s vision. Stromal cells are induced to differentiate into myofibroblasts due to the initial damage of the corneal stroma and corneal epithelial basement membrane. Therefore, stromal cells cannot perform normal functions until the epithelial layer is fully repaired. Col-EDC-PRA gradually regained transparency during the corneal remodeling process. This demonstrated that stromal cells had remodeled most of the allogeneic material. Eventually, all implants will be restored to the normal corneal structure.

Histologic sections stained with H&E provided further insights into the regenerated cornea approximately 5 months after the Col-EDC-PRA implantation (Figure 4b). The sections showed well-organized corneal morphology, with a clearly stratified epithelium with normal squamous epithelial cells and a lamellar arrangement of the stroma. Furthermore, the Col-EDC-PRA membranes seamlessly blended into the stroma, indicating a gradual yet active remodeling process over time. Meanwhile, a histological examination of tissue sections from Col-EDC-PRA implants revealed normal epithelial stratification and the presence of stromal cells within the material. This indicates the growth of corneal stromal cells into collagen patches, promoting corneal stromal regeneration. These dual-crosslinked Col-EDC-PRA membranes demonstrate the potential to fulfill the requirements of corneal implants and offer promising prospects as a regenerative material for focal corneal regeneration.

## 5. Conclusions

In conclusion, the double crosslinked Col-EDC-PRA membranes exhibited superior mechanical properties, suture resistance, optical properties, water content, and enzyme tolerance compared to Col-EDC. The in vivo lamellar keratoplasty results demonstrated that Col-EDC-PRA not only withstood tight suturing on rabbit corneas but also facilitated the remodeling of the corneal epithelium and stroma due to its excellent cell adhesion, migration, and proliferation capabilities. These findings indicate that Col-EDC-PRA is a promising tool for anterior corneal lamellar injury treatments that holds significant potential for future clinical applications.

By accelerating re-epithelialization and corneal regeneration, this novel membrane offers a potential solution to the global shortage of donor corneas. Advancing our understanding of collagen-based membrane applications in ocular reconstruction brings us closer to successful corneal transplantation and the eventual restoration of a structurally and functionally normal ocular surface.

## Data Availability

Not applicable.
